# Nonlinear Influence of Chinese Real Estate Development on Environmental Pollution: New Evidence from Spatial Econometric Model

**DOI:** 10.3390/ijerph19010588

**Published:** 2022-01-05

**Authors:** Wenqin Gong, Yu Kong

**Affiliations:** 1 School of Public Affairs, Chongqing University, Chongqing 400044, China; gongwenqin@cqu.edu.cn; 2 Center for Public Economy & Public Policy, Chongqing University, Chongqing 400044, China

**Keywords:** real estate development, environmental pollution, entropy method, mediation effect, Spatial Durbin Model, Spatial Mediation Model

## Abstract

Environmental pollution is a problem of universal concern throughout the globe. The development of real estate industry not only consumes huge resources, but also has close ties with high-consumption industries such as the construction industry. However, previous studies have rarely explored the impact of real estate development on environmental pollution. Therefore, this paper employs the entropy method to construct a comprehensive index of environmental pollution based on panel data of 31 provinces in China from 2000 to 2017, and empirically examines the impact of real estate development on environmental pollution. This article uses real estate investment to measure the development of the real estate industry. In view of the high spatial autocorrelation of environmental pollution, this paper selects a spatial econometric model. The empirical study found that: (1) By using the Spatial Durbin Model, real estate development has an inverted U-shaped impact on environmental pollution. Meanwhile, most cities have not yet reached the turning point; that is, with the continuous development of the real estate industry, environmental pollution will continue to increase. (2) Further regional heterogeneity found that the inverted U-shaped relationship still exists in coastal and inland areas. (3) Finally, this article used the Spatial Mediation Model to explain the nonlinear impact of real estate development on environmental pollution, with two important mediating variables: population density and industrial structure. Through the above analysis, it can be observed that real estate development has a significant impact on environmental pollution. Thus, the country and the government can reduce environmental pollution by improving the investment structure, using environmentally friendly building materials, guiding population flow and promoting industrial upgrading.

## 1. Introduction

Since the 1990s, environmental problems have been the main agenda of international platforms. Environmental pollution also threatens the sustainability of human life [1]. As a rapidly developing country, China is facing particularly serious environmental problems. Since the reform and opening of China, it has achieved rapid economic growth through the development mode of “environment for growth”, but the extensive growth mode has caused serious environmental pollution [2]. According to the development report of the World Bank in 2001, China is among the top 16 most polluted countries in the world [3]. In 2015, the air quality of 265 of 338 cities above prefecture level exceeded the standard, which was very poor. In 2016, 214 large- and medium-sized cities generated 1.48 billion tons of general industrial solid waste [4].

Activity in the real estate industry is often considered to be the main cause of many environmental problems ranging from non-renewable resource consumption, to the smog clouds, to global warming [5]. In the European Union, about 40% of social energy consumption comes from the real estate industry [6]. Globally, the construction sector contributes up to 30% of carbon emissions, of which the housing sector accounts for 24.5% of this sector [7].

The environmental problems of the real estate industry are more prominent in developing countries [8]. The reform of China’s real estate market in 1998 promoted the vigorous development of the real estate industry. However, China’s real estate industry has neglected the issue of environmental protection in the development process. In order to maximize the pursuit of their own interests, many real estate developers will choose to destroy the ecological environment, leading to barren mountains in many cities in China. In some large cities, building density and volume are too high, and reinforced concrete forests even appear in some places, which means that there is a lack of green space in cities and serious pollution in urban areas [9]. Since 2014, China has become the world’s largest carbon emitter, accounting for about a quarter of global carbon emissions [10]. According to data from the China Building Energy and Carbon Emissions Database, the energy consumption of China’s civil construction sector reached 857 million tons of standard coal equivalent in 2015, accounting for 19.93% of the total energy consumption [11]. 

In recent years, the concept of “green housing” has appeared in China’s housing policy agenda and has become the dominant trend in China’s new housing construction [12,13]. China promotes the construction of green real estate in order to achieve environmental protection, energy saving, and emission reduction goals required by green real estate [14]. According to estimates by Balaras et al., the use of green wall materials and energy-efficient window products can help residential buildings save 33–60% and 14–20% of operating energy consumption, respectively [15]. Galante and Pasetti estimate that if the existing housing is retrofitted with energy-efficient wall materials, the residential construction industry will save 24.8% of energy [6].

In the past, many scholars have studied the impact of the construction industry on environmental pollution, but there are few articles on the impact of real estate on environmental pollution. However, in the process of Chinese rapid economic development for more than 30 years, the status of the real estate industry as a Chinese pillar in the national economy has become more and more important. The ratio of completed investment in real estate development to GDP increased from 4.31% in 1998 to 14.02% in 2020, with an average annual growth rate of 5.5% [16]. Among the 19 industries included in the statistics, the real estate industry was rated second for its volume of fixed asset investment, preceded only by the manufacturing industry [17]. Since China’s real estate market reform was carried out in 1998, China’s real estate industry has developed by leaps and bounds. 

Therefore, this article wants to know how real estate development affects environmental pollution. What mechanism does this influence carry on? How to reduce environmental pollution while promoting real estate development? 

This paper attempts to investigate the impact of real estate development on environmental pollution and its mechanisms by using the panel data of 31 provinces in China from 2000 to 2017.

Gerdesmeier et al. (2010) found that investment has a profound impact on the development of the real estate industry, and the scale of investment changes can be used to predict the rise and fall of real estate [18,19]. Therefore, this article uses real estate investment to measure the degree of real estate development.

**Figure 1:**
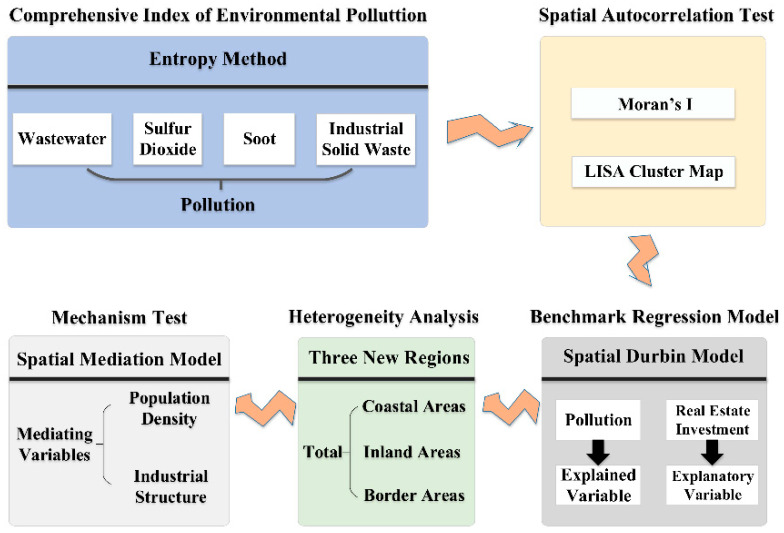
Analysis framework.

The research structure is shown in Figure 1. First, we use the entropy method to construct a comprehensive index to measure regional environmental pollution and use the Moran’s I index and LISA cluster map to investigate the spatial correlation of environmental pollution. Second, we empirically analyze the nonlinear impact of real estate development on environmental pollution through the spatial Durbin model. Further, China is divided into coastal, inland and border areas to examine regional heterogeneity. Finally, in order to study the transmission mechanism of the impact of real estate development on environmental pollution, we use the mediation effect to construct a spatial mediation model.

We conclude that: (1) With the development of real estate, environmental pollution will first increase and then decrease, which shows an inverted U-curve, and most cities are still in the stage of increase. (2) Further analysis of regional heterogeneity shows that this inverted U-curve is mainly reflected in coastal areas and inland areas. (3) Through theoretical and empirical analyses, it can be observed that the nonlinear impact of real estate development on environmental pollution is realized through two mediating variables: population density and industrial structure. Thus, the country and the government can reduce environmental pollution by improving the investment structure, using environmentally friendly building materials, guiding population flow, and promoting industrial upgrading.

## 2. Literature Review

### 2.1. Factors Affecting Environmental Pollution

As for the problem of environmental pollution, the existing research mainly focuses on the causes, mechanism, path and strategies, and means of environmental pollution control. Most studies discuss the cause and effect of environmental pollution from the perspective of natural science [20,21,22]. In recent years, some scholars have begun to explore the institutional causes of environmental pollution from the perspective of social science. These studies use qualitative analysis to focus on the systems, means, and strategies to reduce environmental pollution [23,24]. At the same time, some scholars empirically analyze the socio-economic basis of reducing environmental pollution by using the available environmental pollution data. Among them, most studies are based on many key factors affecting environmental pollution, and they study their path and impact mechanism on environmental pollution. The context of the studies refers to economic growth [25,26,27], foreign trade [28,29,30,31,32], industrial structure [33], financial development level [34], traffic conditions [35], etc.

However, studies on the impact of real estate development on environmental pollution are relatively scarce. Early research mainly focused on the relationship between the construction industry and environmental pollution [36,37,38,39,40]. The construction industry is the world’s largest energy consumer and producer of greenhouse gases [41,42,43], which has caused a huge burden on the environment. The construction industry includes a series of components such as residential, commercial buildings, transportation, government and military facilities, roads, and bridges. Due to its huge scale, it is one of the largest users of energy, material resources, and water [44,45]. In the United States, buildings consume about 74% of total electricity consumption [46]. Although the development of the construction industry can promote economic growth, the contribution of the construction industry to the economy is actually at the cost of the increase of pollution level due to its huge consumption of energy and a series of pollutants [47,48].

### 2.2. The Relationship between Real Estate and Construction

The construction industry is closely related to the real estate industry [49,50]. The construction industry is not only an important supplier but is also a user of the real estate industry [51]. Construction is a common activity for real estate development [52], and the real estate industry provides services for the construction industry [53]. Classified by purpose, real estate includes residential building, office building, commercial building, and other buildings [16]. These types of buildings are all important components of the construction sector [54]. As far as Italy is concerned, residential construction investment accounts for approximately 50% of the entire construction industry and more than 30% of national investment [55]. In China, the development of commercial and residential buildings is the main driving force of rapid urbanization. In order to meet the urgent needs of the growing urban population, Chinese cities have to use energy-intensive concrete to build buildings [56]. This has caused a huge burden on the environment and resources.

### 2.3. Relationship between Real Estate Development and Environmental Pollution

The existing literature studies on the relationship between real estate development and environmental pollution are mainly from the perspective of real estate investment, and most of the literature is analyzed from a qualitative perspective.

Some scholars describe a synergistic effect between real estate investment and environmental pollution. The consensus view holds that real estate investment has promoted economic growth, but the increase of environmental pollution is inevitable in this process [57,58,59]. However, other scholars find that there exist periodic characteristics between real estate investment and environmental pollution. With the increase of real estate investment, the level of environmental pollution will show an inverted “U” relationship [60]. Since real estate investment has rapidly stimulated economic growth in the early stage of economic development [61,62], environmental problems caused by the rapid development of industrialization and urbanization are easy to ignore [63,64,65]. However, with sustained economic development, the society and the government pay more attention to the importance of green development, and the individual’s awareness of environmental protection is gradually strengthened. In order to reduce environmental pollution, attention has been paid to the development of green technology [66,67]. Further, Chen and Lee studied the impact and mechanism of real estate investment on PM_2.5_ in China. They found that the inverted “U” relationship between them can be explained by urban greening coverage, population density and industrial structure [68].

In summary, existing research enriches our understanding of the relationship between real estate development and environment pollution. However, we also found the following deficiencies. First, most literature used only single indicators such as carbon emission [69,70], SO_2_ [71], PM_2.5_ [72,73] to measure environmental pollution, but a single indicator cannot objectively and comprehensively evaluate the actual environmental pollution of a region. Second, most of the previous studies adopt the method of panel regression, which implicitly assumes that there is no correlation between adjacent regions. There are many social and economic connections between regions. Researchers should consider exploring the spatial impact of real estate development on environmental pollution. Therefore, we assume that real estate development will not only affect the environmental pollution in local areas, but also the environmental pollution in adjacent areas. Thus, this paper uses the entropy method to build a comprehensive index of environmental pollution, which can more comprehensively evaluate the environmental pollution situation. Besides, considering the strong spatial spillover of environmental pollution, this paper uses the Spatial Durbin Model and Spatial Mediation Model to study the impact of real estate development on environmental pollution and its transmission mechanisms.

## 3. Model Construction and Variable Selection

### 3.1. Comprehensive Index of Environmental Pollution

In order to reflect on the Chinese environmental pollution situation, we thought about constructing a comprehensive index of environmental pollution. According to Li (2019) and Zhao (2019), we selected wastewater emission, SO_2_ emission, soot emission, and industrial solid production of 31 provinces from 2000 to 2017 as measurement indicators by using the entropy method [74,75]. The relevant data are mainly from China Statistical Yearbook (2001–2018).

The specific construction process is:

First, standardize the original data. Let *y_ij_* represent the value after dimensionless treatment, *x_ij_* is the original value of pollution, *i* represents the province and *j* represents the pollutant,

(1) $$y_{i}{}_{j}=\frac{x_{i}{}_{j}-x_{j\min}}{x_{j\max}-x_{j\min}}(i=1,2\ldots 31,j=1,2,3,4)$$

The second step is to define standardization, including:

(2) $$Y_{ij}=\frac{y_{ij}}{\sum_{j=1}^{m}y_{ij}},\;\mathrm{where}\;m=31$$

where *Y_ij_* represents the environmental pollution index after standardization.

The third step is to calculate the entropy *e* and information utility *d* of the index information, which are:

(3) $$e_{j}=-\frac{1}{\ln m}\sum_{i=1}^{m}Y_{i}{}_{j}\ln Y_{ij}$$

(4) $$d_{j}=1-e_{j}$$

Then, calculate the weight of each pollution, including:

(5) $$W_{j}=\frac{d_{j}}{\sum_{j=1}^{n}d_{j}},\;\mathrm{where}\;n=4$$

where *W_j_* represents the weight of the *j*-th pollution index. It can be seen that the greater the information utility value, the greater the weight of the index.

Finally, the comprehensive index of environmental pollution after assignment is calculated, including:

(6) $${\mathrm{Pollution}}_{i}{}_{j}=\sum_{j=1}^{4}W_{j}y_{i}{}_{j}*100$$

where Pollution represents the comprehensive index of environmental pollution calculated by weighting unused indexes with the entropy weight method. The larger the index value is, the more serious the pollution degree.

### 3.2. Spatial Autocorrelation Test

#### 3.2.1. Moran’s I Analysis

Spatial correlation analysis should be carried out by constructing a spatial weight matrix. We usually use global Moran’s I to measure spatial correlation. The value of Moran’s I ranges from −1 to 1. If Moran’s I is greater than 0, it indicates that there is spatial positive autocorrelation; if Moran’s I is less than 0, it indicates that there is spatial negative autocorrelation; if Moran’s I is close to 0, it means that the spatial distribution is random and that there is no spatial autocorrelation.

The calculation formula of Moran’s I is as follows:

(7) $$I=\frac{\sum_{i=1}^{n}\sum_{j=1}^{n}w_{ij}(x_{i}-\bar{x})(x_{j}-\bar{x})}{S^{2}\sum_{i=1}^{n}\sum_{j=1}^{n}w_{ij}}$$

where $S^{2}=\frac{\sum_{i=1}^{n}{\left(x_{i}-\bar{x}\right)}^{2}}{n}$ represents sample variance, which stands for spatial weight matrix, and adjacency matrix is used to measure the spatial weight matrix.

From Table 1, it can be observed that the Moran’s I of the comprehensive environmental pollution index calculated by Stata 15.0 (Stata, College Station, TX, USA) shows that the values from 2000 to 2017 are greater than 0 and significant at the level of 10%, indicating that there is a spatial positive autocorrelation of environmental pollution in Chinese provinces during this period. We can also see that there were two special values in 2008 and 2013, respectively.

#### 3.2.2. LISA Cluster Map

Moran’s I can only investigate the agglomeration of environmental pollution in the whole space, but it cannot clearly reflect the spatial agglomeration near a certain region. LISA cluster map is used to reflect the local spatial correlation of environmental pollution. High–High (HH) cluster means that the high environmental pollution area is surrounded by the same high environmental pollution areas; Low–Low (LL) cluster means that the area of low pollution environment area is surrounded by the areas of low pollution environment; Low–High (LH) agglomeration means that the area with low environmental pollution is surrounded by the areas with high environmental pollution; High–Low (HL) agglomeration indicates that areas with high environmental pollution are surrounded by areas with low environmental pollution.

**Figure 2:**
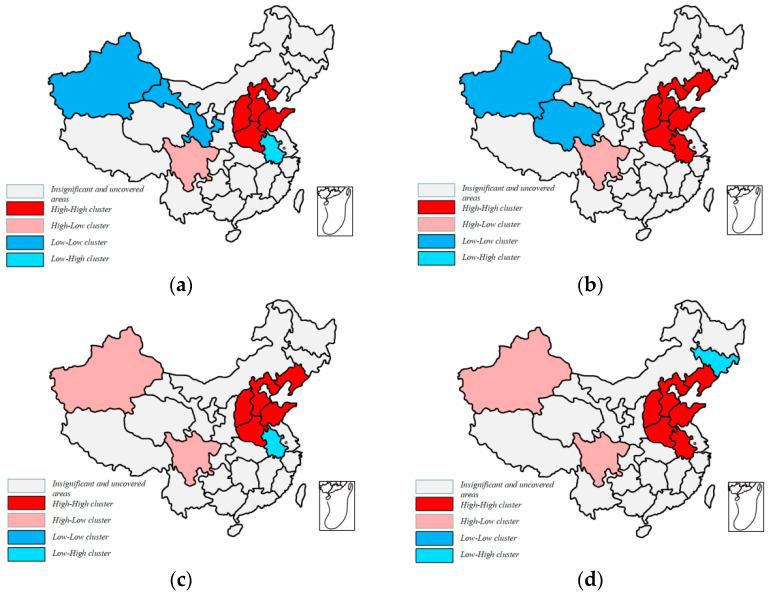
LISA cluster map of pollution in China for the years: (**a**) 2000; (**b**) 2008; (**c**) 2013; (**d**) 2017. (Note: The LISA cluster map of environmental pollution was analyzed by Geoda (Dr. Luc Anselin) software.).

Figure 2 shows the local spatial agglomeration trend of Chinese environmental pollution values at the 10% significance level in four representative years: 2000, 2008, 2013, and 2017. From the charts, it shows that the eastern coastal areas are mainly HH clusters, and with the trend of time, this agglomeration has a trend of proliferation. The western region changed from LL agglomeration in the early stage to HL agglomeration. This shows that there is a certain spatial correlation between environmental pollution among provinces in China.

### 3.3. Variable Description and Data Sources

The explained variable is environmental pollution. Since real estate investment is a representative indicator of real estate development, the core explanatory variable of this article is represented by real estate investment [76], and the square term of real estate investment is introduced to measure the nonlinear impact of real estate development on environmental pollution [68].

This paper also introduces a series of control variables, including:

Economic growth (pgdp) is an important factor affecting environmental pollution. Friedl and Getzner and Shao believe that the three terms brought into the economic level are more suitable to reflect the relationship between economic growth and environmental pollution [77,78]. Therefore, this paper introduces the primary, secondary, and tertiary terms of economic growth. We select per capita GDP to measure economic growth, which takes 2000 as the base year to obtain the actual per capita GDP of that year and to exclude the impact of inflation. 

Opening to the outside world (open) is another important variable affecting environmental pollution. The proportion of total imports and exports in GDP of each region is introduced to measure the degree of opening to the outside world [74]. 

Public transport (bus) is necessary to reduce pollution. With the process of urbanization, automobile exhaust emission has gradually become one of the most important sources of environmental pollution. By actively using public transport and building a low-carbon transportation system, we can reduce road congestion and the dependence on private cars, in order to reduce exhaust emissions and environmental pollution [79]. This paper uses the ownership of public transport vehicles per 10,000 people to measure the degree of public transport [80]. 

The impact of financial development (finance) on environmental pollution is also of great concern. Many scholars believe that the development of financial markets cannot reduce pollution. On the contrary, it will lead to an increase of economic activities and thus increase energy consumption. Therefore, it is one of the driving forces for the increase of pollution [81,82,83]. According to Wei and Kong, this paper uses the proportion of loan balance of financial institutions in GDP to measure the degree of financial development [57]. 

The higher the level of education (edu), the stronger the people’s awareness of environmental protection, which will urge the government to improve the environment and reduce pollution [60]. We use the number of full-time teachers in colleges and universities per 10,000 people to measure people’s education level [84]. 

Government activities (gover) are measured by the proportion of the total fiscal expenditure of local governments to GDP.

This paper selects two mediating variables, in which population density (popden) is measured by the proportion of population and area of each province; the industrial structure (indus) is measured by the ratio of the added value of the secondary industry to GDP. The specific calculation method of each variable is shown in Table 2.

This paper selects 31 provinces in China from 2000 to 2017 as the research object, and the relevant data are mainly from China National Bureau of statistics, China Statistical Yearbook, China Environmental Statistical Yearbook and EPS database. The individual missing values are interpolated by linear interpolation method.

### 3.4. Benchmark Regression Model: Spatial Durbin Model

Through the previous analysis, it can be observed that in the process of real estate development, it consumes considerable energy, material resources, and water and will produce a series of pollutants such as noise, dust, and solid waste, which will directly aggravate environmental pollution. However, as real estate development promotes economic development; society will pay more attention to the importance of green development. Therefore, green technology will be introduced into real estate development to improve efficiency and reduce energy consumption, such as to reduce environmental pollution. Therefore, we assume that the development of real estate has a non-linear impact on environmental pollution, that is, during the development of real estate, environmental pollution presents a trend of increasing first and then decreasing.

In order to test the impact of real estate development on environmental pollution, we constructed the following panel model:

(8) $$\ln{\mathrm{pollution}}_{it}=\alpha +\beta_{0}\ln{\mathrm{realestate}}_{it}+\beta_{1}\ln{\mathrm{realestate}}^{2}{}_{it}+\beta \ln X_{it}+\varepsilon_{it}$$

where *i* represents the province, *t* represents the year, *α* is a constant term, *β* is the correlation coefficient, and *ε* is the error term; pollution represents environmental pollution; realestate represents real estate development. The rest are control variables, represented by *X*, including economic growth (pgdp); public transport (bus); opening to the outside world (open); government activities (gover); financial development (finance); education level (edu). All variables are treated with a logarithm to reduce heteroscedasticity.

We constructed a spatial econometric model to study the direct and spillover effects of real estate investment on environmental pollution. The spatial econometric model generally includes the Spatial Lag Model (SLM), Spatial Error Model (SEM), and Spatial Durbin Model (SDM).

SLM indicates that there is spatial correlation between the explained variables in adjacent areas; SEM considers that the perturbation term ε has spatial dependence. If the explained variables of a region are correlated with the explanatory variables of the local region and adjacent regions, we should build SDM.

The model is selected through a series of tests. LM test is based on the residual of OLS model to verify whether there is a spatial effect in the model. The original hypothesis of this test is that there is no spatial effect in the model. The LM test results showed that the SEM test did not pass the test with significance of 0.05, but the SLM test passed the test with significance of 0.05. As the first mock exam is concerned, Elhorst (2010) has pointed out that in the LM Test, SDM should be constructed as long as one model in SEM and SLM passes the significance test [85]. Then, the Hausmann test is performed on the SDM to choose a fixed-effects model or a random-effects model. The results of the Hausman test are significant. Therefore, the fixed effect model should be selected. Next, LR test is performed to check whether SDM can degenerate into SLM or SEM. From Table 3, it can be observed that the results are significant. We can draw a conclusion that SDM cannot degenerate into SEM or SLM. Otherwise, we examine whether the model has time or individual effects.

Through the above tests, we empirically concluded that the Spatial Durbin Model fits better with reality. This is also in line with our theoretical hypothesis that real estate development can not only affect the environmental pollution level of the local area but also the environmental pollution level of neighboring areas. Finally, this paper constructs the Spatial Durbin Model with time effect:

(9) $$\begin{array}{l} \ln{\mathrm{pollution}}_{it}=\alpha +\delta W\ln{\mathrm{pollution}}_{it}+\beta_{0}\ln{\mathrm{realestate}}_{it}+\beta_{1}\ln{\mathrm{realestate}}^{2}{}_{it}\\ +\beta \ln X_{it}+\theta_{0}W\ln{\mathrm{realestate}}_{it}+\theta_{1}W\ln{\mathrm{realestate}}^{2}{}_{it}+\theta W\ln X_{it}+v_{i}+\varepsilon_{it} \end{array}$$

(10) $$\varepsilon_{it}=\varphi W\varepsilon_{it}+u_{it}$$

where *v_i_* represents for time effect.

## 4. Empirical Results

### 4.1. Regression Results of SDM

Table 4 shows the regression results of SDM. Main represents the regression coefficient *β*; WX represents the regression coefficient *θ*. Because the two columns of regression results adopt the method of point estimation, Lesage and Pace (2009) believe that there are some errors in the conclusion via point estimation in the spatial regression model. According to the different influence of sources of independent variables on dependent variables, Lesage and Pace believe that the coefficient estimation should be decomposed into direct effects and spillover effects by using the partial differentiation method [86].

From the perspective of direct effect, the relationship between real estate development and environmental pollution shows an inverted “U” shape. That is, with the development of real estate, the environmental pollution first rises, and after reaching the critical value, the local pollution level decreases. The conclusion is consistent with the conclusions studied by other scholars [60,68]. If the primary and secondary coefficients of real estate development are a_1_ and a_2_, respectively, the critical value is lnrealestate = −a_1_/2a_2_ = 3.188, and the average value of real estate development is 2.188. The value in 2017 is basically less than 3.188, which shows that the real estate investment of most cities is on the left side of the inverted U-shaped curve. Therefore, the development of real estate at this stage will promote the increase of local environmental pollution. However, from the perspective of a spatial spillover effect, the secondary term of real estate development is positive but not significant, indicating that the environmental pollution of adjacent areas caused by real estate investment shows an insignificant “U” curve trend. Although this “U” curve is not statistically significant, it still shows that, with the local real estate investment increasing, the level of environmental pollution in adjacent areas will first decline and then rise. The increase of real estate investment promotes economic development and attracts more people to enter the region. The outflow of population leads to the reduction of resource consumption, thus reducing the environmental pollution in adjacent regions. However, with the continuous increase of real estate investment at the local region, the high house prices make the floating population unable to bear, thus reducing the attraction of the region. The population of adjacent areas increases, and the consumption of resources increases, thus increasing environmental pollution.

The cubic term coefficient of economic growth on local environmental pollution is positive and significant, indicating that the relationship between economic growth and environmental pollution at the local region presents an inverted “N” curve. If *b*_1_, *b*_2_, and *b*_3_ are used to represent the coefficients respectively, the two critical values of economic growth can be calculated:

$$\ln\mathrm{pgdp}=\frac{-2b_{2}\pm \sqrt{4b_{2}^{2}-12b_{1}b_{3}}}{6b_{3}}.$$

It can be calculated from the formula that the two critical values are 9.15 and 10.805, and the average value of economic growth is 9.136. Most values of economic growth in 2017 have exceeded the first critical value, but all are lower than the second critical value, indicating that most cities have crossed the first critical value; with the development of economy, the level of environmental pollution is increasing. The spillover effect of economic growth on adjacent areas is an insignificant “N” curve.

Both the direct effect and spillover effect of opening to the outside world are negative, indicating that environmental pollution decreases with an increase of opening level. This is because opening can introduce more advanced technologies from abroad to improve production efficiency [87] and change the inefficient and extensive production mode, i.e. reducing environmental pollution [83].

The direct effect of government activities is negative, and the spatial spillover effect is positive. This shows that with an increase of government expenditure, it is helpful to the reduce environmental pollution in the local region, but it will lead to an increase of environmental pollution in adjacent areas. Under the green development mode, the local government has stricter environmental regulations. It is beneficial to reduce local environmental pollution, but it is not conducive to the survival and development of polluting enterprises. Therefore, they will transfer to the adjacent areas with lower environmental regulations, thus increasing environmental pollution in the adjacent areas.

The direct effect of financial development is positive, and the spatial spillover effect is negative. With the development of a financial market, environmental pollution shows an increasing trend in local region, while environmental pollution shows a downward trend in adjacent regions. That is because the development of a financial market promotes an increase of economic activities and attracts the industries and population of adjacent areas into the region [88,89]. However, economic activities promote the emission of pollutants from energy consumption, thus increasing local pollution level and reducing the pollution level of adjacent areas.

The direct effect and spatial spillover effect of education level are negative. The higher the people’s education level is, the stronger the green consciousness they have. This can promote the green development of the economy and reduce environmental pollution. 

The direct effect and spatial spillover effect of public transport are negative. Convenient public transport is conducive to reducing urban congestion and exhaust emissions. Otherwise, local public transport can promote the development of public transport in adjacent areas. Therefore, the development of public transport is conducive to reducing environmental pollution in local region and adjacent areas.

### 4.2. Heterogeneity Analysis

In order to explore regional heterogeneity, the provinces in China are divided into regions for empirical analysis. The traditional approach is to divide China into three regions according to regional characteristics: East, Middle, and West. This division is mainly based on the characteristics of weak external relations in the early stage of reform and opening up, but it can no longer reflect the connection characteristics of today’s provinces in China. It ignores the biggest difference in the level of regional economic development. For example, the “homogeneity” of Chongqing, Sichuan and central cities in the west is significantly greater than the “heterogeneity”. Furthermore, it cannot reflect the differences within each region, and it separates some integral regions with close internal ties [90]. According to Zhang, this paper subdivides Chinese provinces into “three new regions” based on the main problems, policy orientation, and strategic focus of regional economic development, and the division results are shown in Table 5.

We conducted an empirical study of each region. Results are shown in Table 6: In coastal areas and inland areas, with the development of real estate, environmental pollution presents an inverted “U” curve relationship of first rising and then falling in the local region, but its spatial spillover effect is not significant. In border areas, the impact of real estate investment on the local environmental pollution is not significant, but it has a significant impact on the environmental pollution level of adjacent areas. In coastal and inland areas, real estate development is one of the main ways to promote economic growth; thus, it has great impact on the environment. The difference of real estate development in adjacent areas is small; thus, its spillover effect is not significant. The border areas are sparsely populated, and the economy is relatively backward. The population agglomeration and economic activities brought by real estate investment are small; thus, the impact on environmental pollution is naturally small. In addition, developed cities among border areas can improve energy efficiency through an agglomeration effect. It is helpful to reduce environmental pollution in adjacent areas.

## 5. Mechanism Test

### 5.1. Theoretical Analysis

Many articles only study the quantitative relationship between real estate development and environmental pollution, and few articles theoretically analyze how the relationship between the two occurs. Through the combination of qualitative and quantitative, this paper studies the transmission mechanism of the impact of real estate development on environmental pollution. 

#### 5.1.1. Industrial Linkage Effect

As an important industry of the national economy, the real estate industry has the characteristics of long industrial chain, high correlation, and strong driving force [91]. The industrial chain of real estate includes more than 50 material production departments such as wood, cement, steel, chemical industry, petroleum, and machinery. Different industries have different degrees of correlation with the real estate industry, among which the real estate industry has an obvious impact on non-metallic mineral property, construction industry, chemical industry, social service industry, commerce, etc. [92]. Relevant research shows that for every 100 million yuan increase in China’s housing investment, the investment in 23 related industries will increase by 147.9 million yuan [93]. The real estate industry has a backward-pulling effect on driving resource-oriented industries such as manufacturing industry and a forward-pushing effect on consumption-oriented industries such as textile industry. It also plays a two-way role in promoting circular related industries, such as the financial, insurance and construction industry [19]. Backward-related industries refer to the supply industries of the real estate industry, while forward-related industries refer to industries that generate demand for the real estate industry. Overall, the real estate industry can not only act on the secondary industry, but also on the tertiary industry [94]. From a dynamic perspective, as time continues, the driving effect of the real estate industry on some service and consumer industries will gradually increase, while the driving effect on raw material supply and production industries will decrease [95]. Therefore, real estate development will affect the industrial structure. 

Studies have shown that the secondary industry mainly includes manufacturing, mining and construction, which are often accompanied by high pollution and high energy consumption. Therefore, the higher the proportion of the secondary industry, the higher the level of environmental pollution will be [96,97,98,99,100]. The development of a tertiary industry can reduce the proportion of traditional high-energy consumption industries, promote the improvement of an urban education level, increase peoples’ demand for quality of life, and have stricter environmental regulations, in order to reduce environmental pollution [101,102].

#### 5.1.2. Population Density Effect

Real estate development will affect environmental pollution by affecting population density. The change of population density is mainly caused by population mobility. Population mobility can be explained by push–pull theory. Bogue (1969) systematically expounded the push–pull theory: The factor conducive to the improvement of living conditions in the inflow area is the pull, while the unfavorable living conditions in the outflow area are the push. Population flow is determined by these two forces [103]. The development of real estate has a great impact on the labor market [104,105,106]. The pull of real estate development on population mobility stems from two aspects. The first is to meet the living needs of residents. The development of the real estate industry has promoted the improvement of surrounding infrastructure such as medical care, education, and transportation, which is conducive to the influx of population [19]. The second is to promote employment. According to the analysis in Section 5.1.1, the development of the real estate market will drive the development of many industries, thereby providing many jobs [107,108,109]. At the same time, a higher per capita investment intensity will promote the population agglomeration in the local region and will have a population siphon effect on the adjacent regions [110]. Conversely, the development of the real estate will lead to a rise in house prices and increase living costs, which will have a crowding out effect due to the negative income effect, thus inhibiting the inflow of labor force [111,112,113].

Some scholars believe that population agglomeration leads to the improvement of energy efficiency, which can reduce the ineffective consumption of energy; thus, it is conducive to reducing environmental pollution [114]. However, more scholars emphasize that the higher the degree of population agglomeration, the greater peoples’ demand, resulting in more consumption of natural resources. Otherwise, it will also lead to more emissions of pollutants such as automobile exhaust, domestic wastewater, solid waste, and light pollution, thus increasing environmental pollution [68,115].

**Figure 3:**
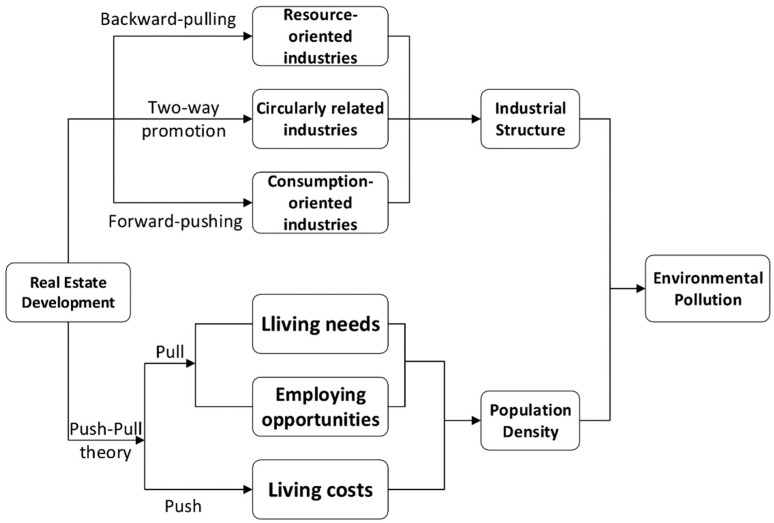
The mechanism of the impact of real estate development on environmental pollution.

To sum up, with the development of real estate investment, industrial structure and population density will be affected. Industrial structure and population density are important factors affecting environmental pollution. The mechanism is shown in Figure 3. Therefore, this article assumes that the development of real estate affects environmental pollution by affecting population density and industrial structure.

### 5.2. Model Construction

This paper uses the nonlinear mediation model [116] to investigate the internal mechanisms of the impact of real estate development on environmental pollution. Referring to Liu and Dong (2021), the spatial mediation model constructed is as follows [117]:

(11) $$\begin{array}{l} \ln M_{it}=\alpha +\beta_{0}\ln{\mathrm{realestate}}_{it}+\beta_{1}\ln{\mathrm{realestate}}^{2}{}_{it}+\beta \ln X_{it}+\theta_{0}W\ln{\mathrm{realestate}}_{it}\\ +\theta_{1}W\ln{\mathrm{realestate}}^{2}{}_{it}+\theta W\ln X_{it}+v_{i}+\varepsilon_{it} \end{array}$$

(12) $$\begin{array}{l} \ln{\mathrm{pollution}}_{it}=\alpha +\delta W\ln{\mathrm{pollution}}_{it}+\rho \ln M_{it}+\beta_{0}\ln{\mathrm{realestate}}_{it}+\beta_{1}\ln{\mathrm{realestate}}^{2}{}_{it}\\ +\beta \ln X_{it}+\xi W\ln M_{it}+\theta_{0}W\ln{\mathrm{realestate}}_{it}+\theta_{1}W\ln{\mathrm{realestate}}^{2}{}_{it}+\theta W\ln X_{it}+v_{i}+\varepsilon_{it} \end{array}$$

where *M* represents the mediating variables, namely industrial structure (indus) and population density (popden); *X* represents a series of control variables, including economic growth (pgdp), openness (open), public transport conditions (bus), government activities (gover), financial development (finance), and education level (edu).

### 5.3. Results and Discussion

When we examine the mediating effect model here, we only consider the main effect of spatial regression (*β*) without analyzing the spatial spillover effect (*θ*). The results are shown in Table 7.

#### 5.3.1. Industrial Structure

From the results in the second column, it is observed that that there is an inverted “U” curve relationship between real estate development and industrial structure. With the development of real estate, the proportion of the secondary industry first increases and then decreases. At the initial stage of real estate development, it is mainly used for housing construction. It consumes various materials such as steel, cement, oil, and machinery. This series of processes has promoted the development of the secondary industry, which increases energy consumption and pollution emissions. However, with the continuous development, peoples’ demand for information transmission, business services, accommodation and catering, finance, education, and other tertiary industries has increased. Their demand for housing quality and green environment has increased, and their awareness of energy conservation and emission reduction also increase, which has promoted the development of the tertiary industry with low energy consumption and green buildings. 

From the results in the third column, we can see that the regression coefficient of industrial structure is positive, indicating that with an increase of the proportion of the secondary industry, the level of environmental pollution also increased. The secondary industry has the characteristics of high pollution. The greater the proportion of the secondary industry, the greater the natural consumption of resources, and the more pollutants are discharged, which will increase environmental pollution. 

To sum up, with the development of real estate, the proportion of the secondary industry first increases and then decreases; thus, the level of environmental pollution also shows a trend of first increasing and then decreasing, which also verifies the conclusion of Chen and Lee [68].

#### 5.3.2. Population Density

From the results in the fourth column, it can be found that the relationship between real estate development and population density also present an inverted “U” curve. With the continuous development of real estate, cities will provide more living needs and employment opportunities, thereby promoting the increase in population density, but at the same time, the cost of living will gradually increase until the floating population cannot afford it, so population density will increase first and then decrease. 

From the regression results in the fifth column, it can be found that the increase of population density will promote the increase of environmental pollution. Human activities have an increasing impact on the environment [118]. Human beings are huge consumers of natural resources [119]. The demand for various high-energy consumption of goods increases, resulting in an increase of environmental pollution [115]. In general, with the development of real estate, the population density first increases, and then decreases; thus, environmental pollution first increases and then decreases.

Generally speaking, the impact of real estate investment on environmental pollution is realized through two ways: population density and industrial structure. This paper also explained the nonlinear relationship between real estate investment and environmental pollution through two ways.

## 6. Conclusions and Policy Implications

### 6.1. Conclusions

Based on the panel data of 31 provinces in China from 2000 to 2017, this paper tested the spatial autocorrelation of environmental pollution by using Moran’s I and LISA cluster map. Then, the Spatial Durbin Model was constructed to test the nonlinear impact of real estate development on environmental pollution. On this basis, regional heterogeneity was considered. Further, by using the two mediation variables of population density and industrial structure, this paper constructed a Spatial Mediation Model to analyze the transmission mechanism of the impact of real estate development on environmental pollution. Finally, the following conclusions were obtained:

First, by calculating the global Moran index and constructing the local LISA cluster map, we found that there is a spatial positive autocorrelation of environmental pollution in China. At the same time, the High–High cluster is significantly concentrated in the eastern coastal area.

Second, there was an inverted U-shaped relationship between real estate development and environmental pollution. However, most provinces in China have not yet reached the transnational critical point. That is, with the development of real estate, most provinces’ environmental pollution will intensify.

Third, this paper examined regional heterogeneity. The inverted “U” relationship between real estate development and environmental pollution could be observed in coastal and inland areas, while the border areas cannot be observed.

Finally, using the Spatial Mediation Model, this research investigated the transmission mechanism of real estate development on environmental pollution. Industrial structure and population density are two important intermediary variables. With the development of real estate, the population density and the proportion of the secondary industry show a trend of first increasing and then decreasing; thus, environmental pollution also shows a trend of first increasing and then decreasing, which can explain the non-linear effects.

Generally speaking, the development of real estate investment will indeed affect environmental pollution. As an important fixed asset, the development and construction of real estate will consume huge resources, which will increase pollution. However, with the rational flow of population, the upgrading of industrial structure and the use of green building materials, environmental pollution will decrease. Thus, there is an inverted U-shaped nonlinear relationship between real estate development and environmental pollution, and this relationship can be explained by two mediation variables, population density, and industrial structure. 

### 6.2. Policy Implications

Through the above conclusions, we believe that we should coordinate the relationship between real estate development and environmental pollution. This paper presents the following suggestions: 

(1) Enterprises should pay attention to the adjustment of industrial structure in order to realize industrial transformation and upgrading. The results show that the higher the proportion of the secondary industry in GDP, the higher the environmental pollution. The secondary industry is often accompanied by high consumption of resources and high emission of pollution. The energy consumption of the tertiary industry is lower, which can help reduce environmental pollution. Since the reform and opening up, China has mainly promoted national economic growth through the development of the secondary industry, which leads to the high proportion of the secondary industry and insufficient development of the tertiary industry. Therefore, both the government and the enterprises should promote the adjustment of industrial structure, pay more attention to innovation of technology, change the traditional development mode of high energy consumption and high pollution, and realize the transformation and upgrading from the secondary industry to the tertiary industry.

(2) Government should reasonably guide population flow and enhance peoples’ awareness of green environmental protection. Population agglomeration is another important factor that aggravates environmental pollution. The eastern coastal area of China is a significant area with a high concentration of pollution. Peoples’ various economic and social activities will aggravate the generation of pollution; however, people can reduce environmental pollution by changing their behavior. Therefore, government needs to properly control the flow of population and curb the disorderly spread of cities and the excessive concentration of population density. Further, government should strengthen green publicity to improve peoples’ awareness of environmental protection. 

(3) The government should formulate and improve policies related to environmental pollution management, promote the construction of an environmental tax, resource tax, and emission trading market, and include the effectiveness of environmental pollution control into the assessment indicators, such as to improve the enthusiasm of the government and enterprises for pollution control.

(4) Society should promote the use of green buildings and energy-saving furniture. Both human destruction of the natural environment and various economic and social activities lead to global warming; thus, various extreme weather and natural disasters are caused. This further promotes peoples’ demand for various consumer goods. Traditional buildings are important factors of pollution in the world. Therefore, society should promote the use of green buildings and energy-saving furniture in order to not only have a higher quality of life for mankind, but also to protect our common home.

Overall, real estate development is an important factor affecting environmental pollution. The government can regulate the relationship between real estate development and environmental pollution by adjusting investment structure, guiding population flow, promoting industrial upgrading, and legislation. Enterprises can reduce pollution by developing energy-saving building materials and by using new technologies to build low-energy houses. Individuals can reduce pollution by improving their awareness of environmental protection, restricting their actions, saving energy, and reducing emissions.

### 6.3. Research Limitations and Future Prospects

This paper studies the impact of real estate investment on environmental pollution and its transmission mechanisms, but there are still many limitations in the research process.

First, due to data limitations, the research object of this paper comprises 31 provinces in China. However, each province includes many cities, which are more closely related to each other. Therefore, studying at the urban level can more truly reflect the relationship between real estate investment and environmental pollution.

Second, the theoretical mechanism analysis of this paper is relatively poor. The impact of real estate investment on environmental pollution and its mechanism are worthy of in-depth discussion in theory. In the past, there were fewer articles studying the impact of real estate development on environmental pollution, and even fewer articles involving theoretical analysis. However, this article finds the mediating variables that affect the relationship between the two through literature review, and analyzes it theoretically and empirically. This analysis can learn from less experience, so the transmission mechanism in theoretical analysis is not perfect, and the selection of mediating variables may be incomplete. However, this provides ideas for future articles, that is, real estate development not only directly affects environmental pollution, but also indirectly affects environmental pollution by affecting other mediating variables.

Third, when investigating the impact mechanisms, this paper does not analyze the impact mechanisms of local real estate investment on environmental pollution in adjacent areas.

Finally, the selection of control variables is not perfect, and there may be the problem of missing variables.

In future research, we can refine the research scope and deepen the theoretical analysis and transmission mechanisms to improve this paper.

## Figures and Tables

**Table 1:** Moran’s I of environmental pollution.

Year	Moran’s I	sd(I)	z	*p* Value
2000	0.222	0.119	2.151	0.016
2001	0.234	0.118	2.255	0.012
2002	0.240	0.118	2.308	0.011
2003	0.240	0.118	2.306	0.011
2004	0.219	0.118	2.140	0.016
2005	0.245	0.119	2.345	0.010
2006	0.259	0.119	2.460	0.007
2007	0.256	0.119	2.441	0.007
2008	0.265	0.118	2.521	0.006
2009	0.259	0.118	2.467	0.007
2010	0.229	0.118	2.218	0.013
2011	0.224	0.116	2.226	0.013
2012	0.204	0.116	2.051	0.020
2013	0.188	0.116	1.905	0.028
2014	0.228	0.116	2.244	0.012
2015	0.258	0.117	2.491	0.006
2016	0.179	0.115	1.845	0.033
2017	0.135	0.118	1.430	0.076

**Table 2:** Variable description.

Variable	Description	Unit
pollution	Wastewater discharge, SO_2_ discharge, soot discharge and industrial solid waste production are constructed by entropy method	/
realestate	Completed real estate investment/GDP	%
pgdp	Real per capita GDP, based on 2000	yuan
open	Total import and export/GDP	%
gover	Local fiscal expenditure/GDP	%
finance	Loan balance of financial institutions/GDP	%
edu	Number of full-time teachers in colleges and universities per 10,000 people	people
bus	Public transport vehicles per 10,000 people	vehicle
popden	Population/area of each province	People/km^2^
indus	Added value of secondary industry/GDP	%

**Table 3:** The result of LR test.

Likelihood-ratio test	LR chi2(10) = 195.4
(Assumption: sar_a nested in sdm_a)	Prob > chi2 = 0.0000
Likelihood-ratio test	LR chi2(10) = 237.91
(Assumption: sem_a nested in sdm_a)	Prob > chi2 = 0.0000

**Table 4:** Regression results of SDM.

Variable	Main	WX	Direct Effect	Spillover Effect
lnrealestate	3.582 ***	−1.005	3.730 ***	−1.726 ***
	(10.708)	(−1.194)	(10.859)	(−2.763)
lnrealestate^2^	−0.567 ***	0.032	−0.585 ***	0.177
	(−6.976)	(0.152)	(−6.981)	(1.061)
lnpgdp	−160.101 ***	−13.380 ***	−157.789 ***	27.685 **
	(−3.600)	(−4.255)	(−3.644)	(2.079)
lnpgdp^2^	16.290 ***	2.977 ***	15.924 ***	−1.511
	(3.405)	(4.653)	(3.430)	(−1.052)
lnpgdp^3^	−0.548 ***	−0.146 ***	−0.532 ***	0.013
	(−3.209)	(−4.325)	(−3.217)	(0.253)
lnopen	−1.022 ***	−0.734 ***	−0.988 ***	−0.325 *
	(−9.932)	(−3.211)	(−9.546)	(−1.950)
lngover	−4.720 ***	2.317 ***	−4.958 ***	3.050 ***
	(−25.878)	(5.639)	(−25.020)	(11.598)
lnfinance	1.722 ***	−1.921 ***	1.874 ***	−2.005 ***
	(6.932)	(−3.746)	(7.539)	(−4.809)
lnedu	−0.194 **	−0.666 ***	−0.150 *	−0.474 **
	(−2.387)	(−2.780)	(−1.900)	(−2.434)
lnbus	−1.894 ***	−2.179 ***	−1.786 ***	−1.270 ***
	(−10.286)	(−5.810)	(−9.659)	(−4.052)

Note: *t* value of the coefficient is in parentheses, ***, ** and * indicate significant at the level of 1%, 5% and 10%, respectively; lnrealestate^2^ represents the quadratic term of lnrealestate.

**Table 5:** Three new regions and their scope.

Coastal areas (10)	Liaoning, Beijing, Tianjin, Hebei, Shandong, Jiangsu, Shanghai, Zhejiang, Fujian, Guangdong
Inland areas (13)	Sichuan, Chongqing, Guizhou, Hubei, Hunan, Anhui, Qinghai, Jiangxi, Gansu, Ningxia, Shaanxi, Shanxi, Henan
Border areas (8)	Heilongjiang, Jilin, Inner Mongolia, Xinjiang, Tibet, Yunnan, Guangxi, Hainan

**Table 6:** Regression results of SDM in each region.

Region	Variable	Direct Effect	Spillover Effect
Coastal areas	lnrealestate	2.195 ***	−1.418
		(3.292)	(−1.105)
	lnrealestate^2^	−0.370 ***	0.201
		(−2.934)	(0.739)
	Control variables	YES	YES
Inland areas	lnrealestate	0.592 ***	−0.451
		(2.717)	(−0.947)
	lnrealestate^2^	−0.260 ***	−0.084
		(−4.913)	(−0.712)
	Control variables	YES	YES
Border areas	lnrealestate	−0.485	−0.938 **
		(−0.893)	(−1.964)
	lnrealestate^2^	0.143	0.168
		(1.082)	(1.013)
	Control variables	YES	YES

Note: *t* value of the coefficient is in parentheses, *** and ** indicate significant at the level of 1% and 5%, respectively; lnrealestate^2^ represents the quadratic term of lnrealestate.

**Table 7:** Regression results of Spatial Mediation Model.

Variable	lnpollution	lnindus	lnpollution	lnpopden	lnpollution
lnrealestate	3.582 ***	0.200 ***	2.899 ***	0.735 ***	3.417 ***
	(10.708)	(5.165)	(8.963)	(3.870)	(10.203)
lnrealestate^2^	−0.567 ***	−0.043 ***	−0.415 ***	−0.218 ***	−0.520 ***
	(−6.976)	(−4.516)	(−5.279)	(−4.727)	(−6.346)
ln*M*			2.808 ***		0.222 ***
			(7.603)		(2.969)
lnpgdp	−160.101 ***	−48.138 ***	−7.983	54.755 **	−168.935 ***
	(−3.600)	(−9.365)	(−0.176)	(2.171)	(−3.818)
lnpgdp^2^	16.290 ***	5.459 ***	−0.950	−6.155 **	17.333 ***
	(3.405)	(9.874)	(−0.193)	(−2.269)	(3.642)
lnpgdp^3^	−0.548 ***	−0.205 ***	0.099	0.226 **	−0.588 ***
	(−3.209)	(−10.382)	(0.557)	(2.337)	(−3.460)
lnopen	−1.022 ***	−0.076 ***	−0.815 ***	0.110 *	−1.053 ***
	(−9.932)	(−6.395)	(−8.038)	(1.879)	(−10.280)
lngover	−4.720 ***	−0.039 *	−4.738 ***	−0.238 **	−4.732 ***
	(−25.878)	(−1.915)	(−26.869)	(−2.360)	(−25.494)
lnfinance	1.722 ***	0.120 ***	1.315 ***	0.058	1.739 ***
	(6.932)	(4.163)	(5.542)	(0.410)	(6.980)
lnedu	−0.194 **	−0.007	−0.153 **	0.023	−0.196 **
	(−2.387)	(−0.712)	(−1.994)	(0.488)	(−2.422)
lnbus	−1.894 ***	−0.142 ***	−1.494 ***	0.633 ***	−2.049 ***
	(−10.286)	(−6.744)	(−8.048)	(6.142)	(−10.916)

Note: *t* value of the coefficient is in parentheses, ***, ** and * indicate significant at the level of 1%, 5% and 10%, respectively; lnrealestate^2^ represents the quadratic term of lnrealestate.

## Data Availability

Data were obtained from the China National Bureau of statistics (https://data.stats.gov.cn/ accessed on 15 August 2021), China Statistical Yearbook (accessed on http://www.stats.gov.cn/tjsj/ndsj/ accessed on 15 August 2021), China Environmental Statistical Yearbook (https://navi.cnki.net/knavi/yearbooks/YHJSD/detail?uniplatform=NZKPT accessed on 15 August 2021), and EPS database (https://www.epsnet.com.cn/index.html#/Index accessed on 15 August 2021).
